# Identification of monotonically expressed long non-coding RNA signatures for breast cancer using variational autoencoders

**DOI:** 10.1371/journal.pone.0289971

**Published:** 2023-08-10

**Authors:** Dongjiao Wang, Ling Gao, Xinliang Gao, Chi Wang, Suyan Tian

**Affiliations:** 1 Department of Gynecological Oncology, The First Hospital of Jilin University, Changchun, Jilin, People’s Republic of China; 2 Department of Radiation Oncology, The First Hospital of Jilin University, Changchun, Jilin, People’s Republic of China; 3 Department of Thoracic Surgery, The First Hospital of Jilin University, Changchun, Jilin, People’s Republic of China; 4 Department of Internal Medicine, College of Medicine, University of Kentucky, Lexington, Kentucky, United States of America; 5 Markey Cancer Center, University of Kentucky, Lexington, KY, United States of America; 6 Division of Clinical Research, The First Hospital of Jilin University, Changchun, Jilin, People’s Republic of China; The First Affiliated Hospital of Nanjing Medical University, CHINA

## Abstract

As breast cancer is a multistage progression disease resulting from a genetic sequence of mutations, understanding the genes whose expression values increase or decrease monotonically across pathologic stages can provide insightful clues about how breast cancer initiates and advances. Utilizing variational autoencoder (VAE) networks in conjunction with traditional statistical testing, we successfully ascertain long non-coding RNAs (lncRNAs) that exhibit monotonically differential expression values in breast cancer. Subsequently, we validate that the identified lncRNAs really present monotonically changed patterns. The proposed procedure identified 248 monotonically decreasing expressed and 115 increasing expressed lncRNAs. They correspond to a total of 65 and 33 genes respectively, which possess unique known gene symbols. Some of them are associated with breast cancer, as suggested by previous studies. Furthermore, enriched pathways by the target mRNAs of these identified lncRNAs include the Wnt signaling pathway, human papillomavirus (HPV) infection, and Rap 1 signaling pathway, which have been shown to play crucial roles in the initiation and development of breast cancer. Additionally, we trained a VAE model using the entire dataset. To assess the effectiveness of the identified lncRNAs, a microarray dataset was employed as the test set. The results obtained from this evaluation were deemed satisfactory. In conclusion, further experimental validation of these lncRNAs with a large-sized study is warranted, and the proposed procedure is highly recommended.

## Introduction

Breast cancer is the most commonly diagnosed cancer and the most frequent cause of cancer death among women worldwide [1]. Since cancer is a multistage progression process resulting from genetic sequence mutations [2], the genes whose expression values increase or decrease monotonically across stages are expected to play essential roles in the tumor progression and metastasis. The identification of these monotonically differentially expressed genes (MEGs) offers valuable insights into the progression of cancer. Several studies have investigated MEGs across stages for a variety of cancer types, such as lung cancer [3, 4], colon cancer [4, 5], and liver cancer [6]. Certainly, breast cancer is also included [7].

**Fig 1:**
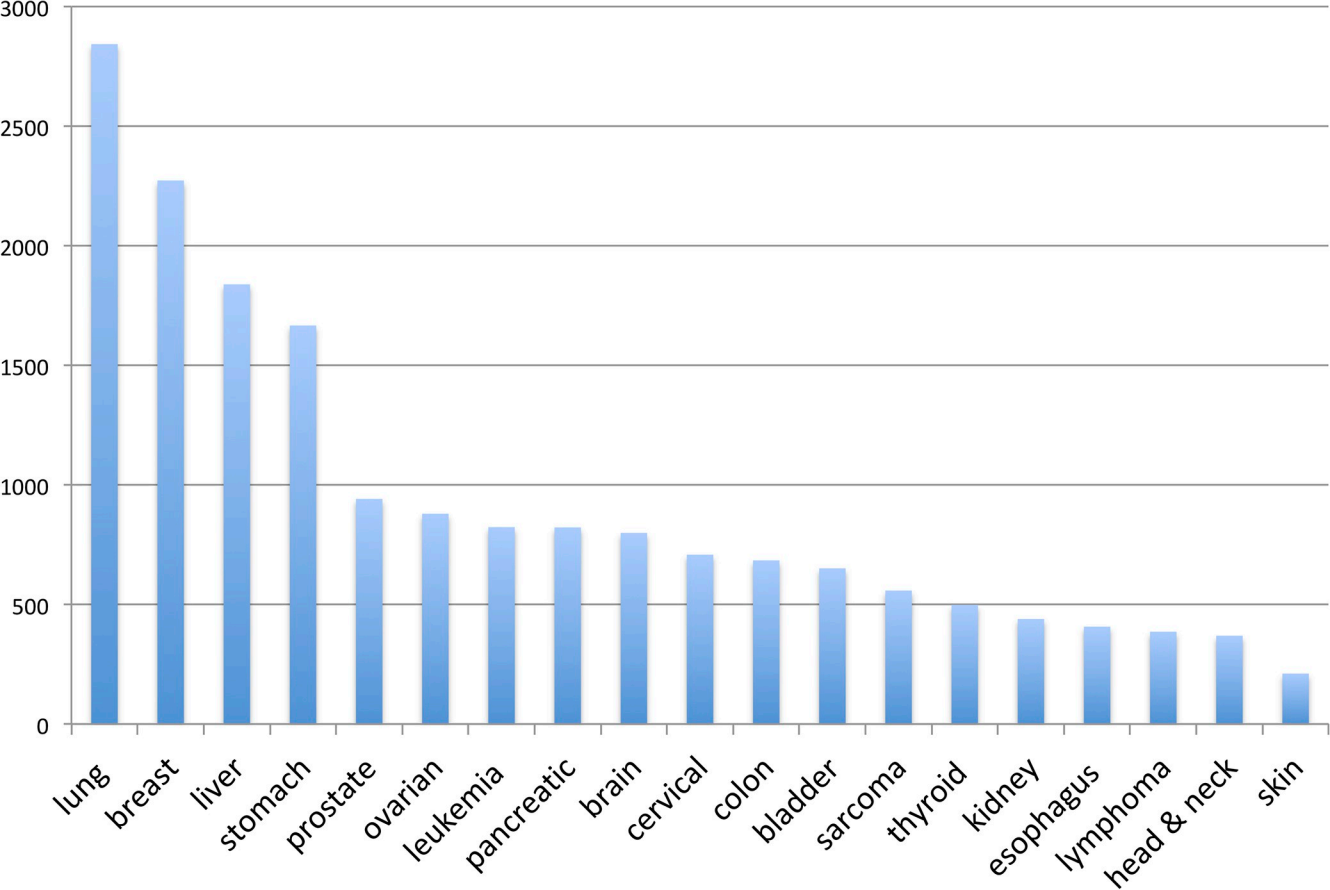
Summary of the PubMed survey searching for the association between long non-coding RNAs and certain cancer types. This survey reveals that breast cancer is the second most frequently studied cancer type in relation to lncRNAs. lncRNAs: long non-coding RNAs.

Long non-coding RNAs (lncRNAs) are post-transcriptional and epigenetic regulators, with usually lower and more tissue-specific expression levels compared to protein-coding genes. Once regarded as evolutionary junk, lncRNAs are now known to play essential roles in complex diseases, including cancer [8]. In respect of breast cancer, deregulation of lncRNAs, such as XIST [9, 10], H19 [11], and HOTAIR [12, 13], has been reported in tumor tissues or cell lines. A survey of the PubMed database using the keywords “lncRNA” and specific cancer types (as depicted in **Fig 1**) revealed that breast cancer ranks as the second most frequently studied cancer type in relation to lncRNAs. This observation highlights the significance of exploring the pivotal role of lncRNAs in breast cancer further.

To our knowledge, no prior study has focused specifically on lncRNAs whose expression values changed monotonically across stages of breast cancer, especially using deep learning methods. Deep learning methods, which are neural networks that comprise multiple layers with non-linear activation functions, have gained immense popularity in many fields, including life science and medicine [14]. Nevertheless, it is worth pointing out that the development and utilization of deep learning methods in the biomedical research face two key obstacles [15, 16], namely the curse of dimensionality and the lack of a good biological interpretation. Deep learning techniques are predominantly employed in the analysis of single cell RNA-sequencing (scRNA-seq) data, which can be considered as “big-big” data due to their large number of cells (n) and features (p). As a result, the problem of over-fitting issue is relatively less severe in scRNA-seq data compared to bulk RNA-seq data and microarray data. The cancer genome atlas (TCGA) breast cancer cohort had enrolled ~1,000 subjects that is relatively large compared to other cohorts, alleviating the curse of dimensionality to a certain extent. Due to this reason, as well as to showcase how to identify and verify MEGs with the aid of a deep learning method, the TCGA breast cancer was chosen.

Furthermore, in contrast to other applications such as computer vision (CV) and neural language processing (NLP), a good biological interpretation is a must for the biomedical research. The “black box” operation of the deep learning methods blurred an explicit interpretation, and thus impeded their widespread utilization in the circles of biology and medicine. Nevertheless, reconstructed gene expression representative values using autoencoder frameworks have previously been shown to reveal novel biological patterns [17, 18]. As anticipated, autoencoder frameworks are expected to unveil monotonically changing patterns as the disease advances. A conventional autoencoder framework comprises two essential components: the encoder and the decoder. First, the data were compressed into a low-dimensional vector representation using the encoder. Next, the low-dimensional vector was uncompressed by the decoder to obtain the reconstructed data to minimize the reconstruction error (i.e., the difference between the original data and the reconstructed data), so the reconstructed data could most closely mimic the original data. Several review articles [19] showed an autoencoder cannot generate new samples, which are not in the original data.

Currently, a variational autoencoder (VAE) model is among the commonest used deep learning methods for modeling gene expression data, taking the work by [20, 21] as an example. Compared to an autoencoder, a VAE is capable of generating representative features that capture the underlying structures of the data, instead of limiting to the original data and “memorizing” them [19]. This task is accomplished by mapping the data into a distribution with a low dimension. The structures being investigated can encompass various phenotypes, cell types, and other characteristics specific to the samples. Therefore, the VAE framework is anticipated to be capable of detecting the specific structure of interest in this study, namely the monotonically changing patterns in expression values as the breast cancer progresses.

In this article, we used the representative expression values learned from VAE models to parameterize monotonically increasing and decreasing lncRNAs, respectively, across pathologic stages of breast cancer and thus justified that the expression levels of identified lncRNAs changed monotonically.

## Materials and methods

### Experimental data

The gene expression profiles and corresponding clinical information of the breast cancer (BRCA) cohort from TCGA were obtained from the TCGA’s Genomic Data Commons (http://www.cbioportal.org/). The cohort comprised 140 patients at stage I, 480 patients at stage II, 180 patients at stage III, and 105 para-carcinoma tissues serving as normal controls, which were included for the downstream analysis. Of note, the patients at stage IV were excluded due to the limited number of individuals (only 14 patients) in this advanced stage within the TCGA BRCA cohort.

The annotation of lncRNAs were retrieved from the TANRIC [22] database, which included 12,727 unique lncRNAs gene symbols. The gene expression profiles were quantified as the fragments per kilo-bases per million (FPKM) counts, then these expression values were subsequently standardized to have means of 0’s and variances of 1’s. For validation purposes, a microarray dataset with 121 individuals included was considered. The expression matrix for this dataset was directly downloaded from the gene expression omnibus (GEO) database under the accession number of GSE42568 (https://ncbi.nlm.nih.gov/gds/?term=GSE42568).

### Identification of monotonically differentially expressed genes

In the proposed procedure of identifying monotonically differentially expressed genes, moderate t-tests (implemented by the R limma package) were conducted to screen out the differentially expressed genes (DEGs); the multiple testing issue was addressed by adjusting p-values with the Benjamini-Hochberg method [23].

As different change patterns, such as “U-shaped”, “waved”, and monotonic patterns, were involved in those DEGs, the following constraints were added in order to extract out the desired monotonic patterns. To investigate the scenario of monotonically decreasing gene expression, we focused on the intersection of three sets of under-expressed DEGs: stage I versus normal, stage II versus normal, stage III versus normal. The assessment of fold change at the logarithmic scale (LFC) was performed. The corresponding restriction is LFC_I_ > LFC_II_ > LFC_III_. In contrast, for the monotonically increasing scenario, LFC_I_ < LFC_II_ < LFC_III_ was additionally required and hence added upon the intersection of over-expressed DEG sets. Since patterns other than monotonic ones are not our focus areas, which may be regarded as “noises”, the inclusion of other patterns would blur the true signals of interest. Therefore, the addition of proposed restrictions should help discard these noises.

### Implementation of VAE models to validate MEGs

A VAE employs a similar strategy to an AE; the only difference between VAE and AE is that in the VAE model, each data point is represented by a set of latent variables that can be decoded by neural networks to produce parameters for a probability distribution (usually a Gaussian distribution). Hence, VAE defines a generative model. In this study, we used a VAE model to justify the identified MEGs in the previous subsection present monotonically changed patterns across stages, indeed.

This procedure is based on the premise that, for each stage, the pattern of all monotonically increasing changed genes (with similar or identical underlying patterns) can be captured by the corresponding VAE model’s underlying distribution. The same rationale is applicable to monotonically decreasing genes as well. Then if the corresponding means of these distributions over stages present monotonic changes (and are statistically significant since we also have the variances of these distributions, hypothesis tests could be carried out. Here, the underlying null hypothesis is μ_normal_ = μ_stage I_ = μ_stage II_ = μ_stage III_), the identified lncRNAs are certainly monotonic.

The encoders of VAE considered in the current study comprised of the following layers: the first dense network connects the input layer to the first hidden layer, the number of nodes is 32, and the second network with 8 nodes. Rectified Linear Unit (ReLU) is the activation functions used in the dense network. Thereafter a hidden layer is connected to the bottleneck layer, which consists of one network for the mean and another network for the variance on the logarithmic scale of the hidden distribution.

**Fig 2:**
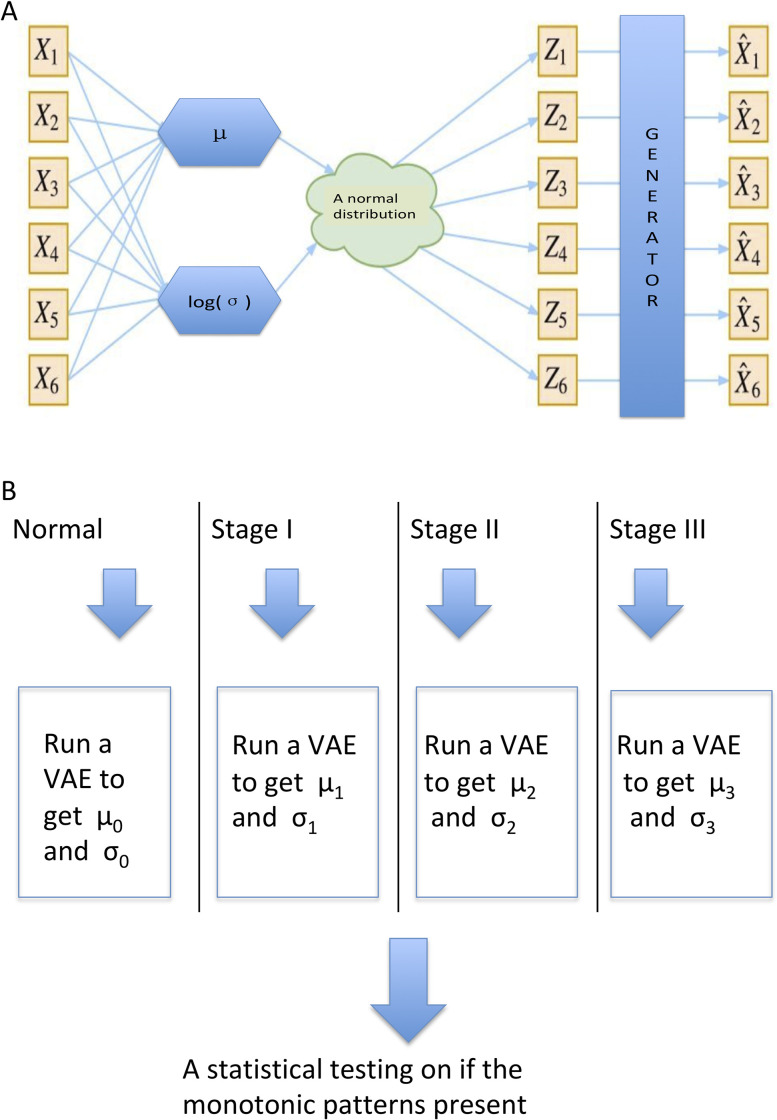
Flowchart illustrating how variational autoencoders were used to justify identified monotonically differentially expressed genes. A. Framework for a VAE model. B. To validate the identified MEGs. VAE: variational autoencoder; MEGs: monotonically differentially expressed genes.

The decoder of VAE comprised of the layers in the inverse order, namely, the first full-connected layer connects the bottleneck layer to an 8-node hidden layer, and the second full-connected layer with 32 nodes, and the output layer decoding back the dimension of original data without an activation function. The Adam method was used as the optimizer, and default settings for the learning rate and other hyperparameters were employed. All VAE modeling was stopped at 20 epochs, which have shown that mean squared error (MSE) metrics in the VAE models converge. **Fig 2** depicts the flowchart of this proposed procedure.

### Biological relevance and enrichment analysis

For the identified monotonic genes, their ensemble gene identifiers were converted to The HUGO Gene Nomenclature Committee (HGNC) approved gene symbols using the GENCODE (https://www.gencodegenes.org/) database (version 38). The GeneCards (https://www.genecards.org) and PubMed databases were searched to survey for the biological relevance of identified monotonically changed lncRNAs with unique gene symbols.

The target mRNAs by the identified lncRNAs were retrieved from the lncRNA Disease 2.0 database [24]. The String software (www.string-db.org) was used to perform pathway enrichment analysis to identify the pathways/gene sets enriched by the target mRNAs.

### Statistical language and packages

R language version 4.0 (www.r-project.org) was used to perform data analyses in this study. To construct VAE models, the Keras package with a TensorFlow backend (with a call on Python language, version 3.7) was employed. Additionally, the limma package was utilized for fitting moderated t-tests.

## Results and discussion

First, the DEGs were identified using moderated t-tests. Setting the cutoff value of the false discovery rate (FDR) at 0.01, for the first comparison in which the patients at stage I and the normal controls were considered, 2,629 lncRNAs were identified as down-regulated DEGs and 838 lncRNAs as up-regulated DEGs, respectively. For the second comparison (stage II versus control), there were 2,966 and 1,205 DEGs, while for the last comparison (stage III versus control), the numbers were 2,970 and 887, respectively. Interestingly, the number of down-regulated DEGs was found to be approximately two times higher than the number of up-regulated DEGs. Then we put additional restrictions (as suggested in the Method section) in order to discard other patterns rather than the monotonic ones. This resulted in 248 monotonically decreasing DEGs and 115 monotonically increasing DEGs, which were input into the VAE models for further justification.

**Fig 3:**
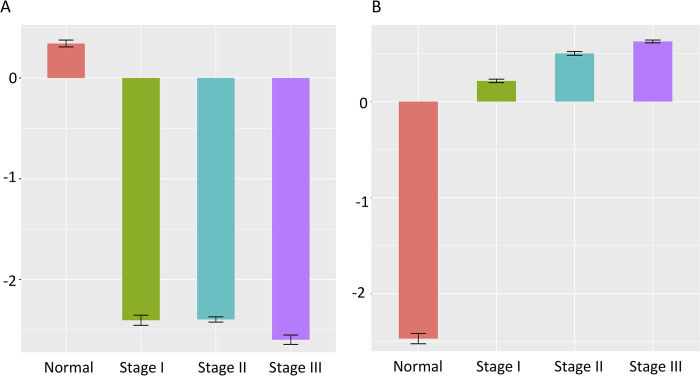
Underlying hidden distributions of VAEs showing the expression values of identified lncRNAs are monotonically changed across stages indeed. A. Decreasing scenario. B. Increasing scenario. All comparisons but stage I versus stage II in the decreasing cases met the level of statistical significance.

The expression profiles of these 248 monotonically decreasing DEGs and 115 monotonically increasing DEGs were fed into the VAE networks (4 VAE models for the decreasing scenarios, including respective individual ones for normal, stage I, stage II and stage III, and the other 4 VAE models for the increasing scenarios, respectively). The representative parameters, namely the mean and variance of underlying latent distribution were extracted. Subsequently, hypothesis testing was performed based on these parameters. **Fig 3** shows that except for the comparison between stage II and stage I in the down-regulated case (p = 0.887), the statistically significant difference between the pairs in the other comparisons was evidence. To conclude, the monotonically changed patterns of identified lncRNAs were obvious.

After transferring the annotation of identified lncRNAs to HUGO gene symbols, the 248 down-regulated and 115 up-regulated MEGs corresponded to 65 and 33 unique lncRNAs, respectively. Out of the 98 identified unique monotonically differentially expressed lncRNAs, 9 were found to be unannotated in the GeneCards database. Among the remaining lncRNAs, For the remaining lncRNAs, 16 were directly associated with breast cancer, meaning previous studies have established a link between these identified gene and breast cancer. Additionally, 44 lncRNAs were indirectly associated with breast cancer, as previous studies have demonstrated that the genes sharing the same biological pathways as the identified genes are associated with breast cancer. In total, approximately 60% of the identified lncRNAs were found to be associated with breast cancer based on the information from the GeneCards database. For instance, using sequencing technique (sequenced on seven pairs of tumor and normal tissues) and bioinformatics methods, LINC00636 was identified as a hub gene in the co-expression network constructed for HER-2-enriched subtype breast cancer [25]. Furthermore, using real-time quantitative polymerase chain reaction (qRT-PCR), MIAT was experimentally validated as being over-expressed in breast cancer patients compared to normal controls [26]. LINC01016 was also experimental validated to associate with breast cancer, saying that this gene’s silencing results in reduced proliferation, and it has a prognostic value on the survival time of breast cancer patients [27]. The regulation direction of MIAT and LINC01016 is in consistent with our results.

Additionally, we explored the potential biological relevance of the target mRNAs by these lncRNAs. The target mRNAs by these 98 lncRNAs were retrieved from the lncRNA Disease 2.0 database. Then the enriched KEGG pathways and GO terms of target mRNAs were obtained using the String software [28] and presented in **Tables 1 & 2**, one corresponding to the increasing scenario and the other to the decreasing scenario. Of the enriched Kyoto Encyclopedia of Genes and Genomes (KEGG) pathways, several pathways such as HIF-1 signaling pathway [29, 30], Wnt signaling pathway [31–33], human papillomavirus infection [34, 35], and Rap 1 signaling pathway [36] have been demonstrated to associate with breast cancer previously. On the other hand, of the enriched GO terms, focal adhesion [37] is relevant to breast cancer, as suggested by the literature. In summary, at the level of pathways, a good biological implication of identified lncRNAs to breast cancer is harnessed.

**Table 1:** Enriched KEGG pathways and GO terms by the target mRNAs of identified monotonically increasing expressed lncRNAs.

ID	Term description	Observed gene count	Background gene count	False discovery rate (FDR)
KEGG pathways				
hsa05211	Renal cell carcinoma	5	66	0.002
hsa05200	Pathways in cancer	10	517	0.0025
hsa04916	Melanogenesis	5	95	0.0035
hsa04066	HIF-1 signaling pathway	5	106	0.0044
hsa04120	Ubiquitin mediated proteolysis	5	135	0.0106
hsa04310	Wnt signaling pathway	5	154	0.0159
hsa05215	Prostate cancer	4	96	0.0246
hsa05165	Human papillomavirus infection	6	325	0.0485
hsa05166	Human T-cell leukemia virus 1 infection	5	211	0.0485
GO cellular component terms				
GO:0030891	VCB complex	3	5	0.0032
GO:0031262	Ndc80 complex	3	4	0.0032
GO:0005667	Transcription regulator complex	9	431	0.0109
GO:0000151	Ubiquitin ligase complex	7	286	0.0254

**Table 2:** Enriched KEGG pathways and GO terms by the target mRNAs of identified monotonically decreasing expressed lncRNAs.

ID	Term description	Observed gene count	Background gene count	False discovery rate (FDR)
KEGG pathway				
hsa03010	Ribosome	7	130	0.0024
hsa04015	Rap1 signaling pathway	7	202	0.018
GO biological process terms				
GO:0009952	Anterior/posterior pattern specification	9	214	0.0278
GO:0003002	Regionalization	10	332	0.0456
GO:0009887	Animal organ morphogenesis	17	967	0.0456
GO:0048706	Embryonic skeletal system development	7	130	0.0456
GO cellular component				
GO:0070161	Anchoring junction	17	820	0.0016
GO:0005925	Focal adhesion	10	405	0.045
GO:0030054	Cell junction	24	2075	0.045
GO:0044391	Ribosomal subunit	7	178	0.045

Finally, to assess the discriminative ability of the identified lncRNAs, gene expression values from the GSE42568 microarray dataset were utilized for validation purposes. This dataset included 17 normal controls, 11 patients at stage I, 40 patients at stage II, and 53 patients at stage III. As the gene annotations differed between the two platforms, we opted to fit an overall VAE model based on the common annotated 40 lncRNAs present in both platforms. The hyperparameters of this overall VAE network were set be identical to those of the separate VAE networks. Overall, 28 patients at different stages were misclassified regarding their histologic stages, resulting in an error rate of 23.14%. Notably, neither normal controls nor breast cancer patients have been misclassified, indicating that the performance of these MEGs is satisfactory.

We acknowledge the present study has several limitations. First, it is well known that breast cancer is a very heterogeneous disease, and different subtypes have district underlying molecular mechanisms and prognoses. The subgroup analyses were not considered in this study given that with a further stratification on subtypes would increase the likelihood of over-fitting dramatically (resulting from the fact that the number of samples in each stratum would drop substantially). Furthermore, it is worth noting that patients at stage IV were excluded in this study due to the limited number of patients at this specific stage (only 14) within the TCGA BRCA cohort. While understanding the progression of breast cancer to this advanced stage is of significant interest, a large-scale study is highly desirable. Additionally, the deep learning model considered in this study, specifically a VAE model with 7 layers can be considered relatively shallow in terms of its complexity. Given that the sample size for each stratum ranges approximately from 100 to 500, such sizes of discovery data are insignificant to train a deep learning model enclosed with a remarkably large number of hidden layers.

## Conclusions

A significant contribution of this study is the ingenious combination of a VAE network, which generates a representation feature representing all genes with a specific change pattern with the classical approach of statistical hypothesis testing. This integrated analysis enables the identification and subsequent validation of monotonically changing genes across different stages of breast cancer. Thus, it enlightens us on a potential stretch for the application of deep learning methods.

Our analysis results show that the identified lncRNAs are indeed changed monotonically across stages and have good biological implications in breast cancer. Experimental validation of these lncRNAs with a large-scale study is warranted in the future. In conclusion, the proposed procedure is highly recommended.

## References

[1] Bray F, Ferlay J, Soerjomataram I. Global Cancer Statistics 2018: GLOBOCAN Estimates of Incidence and Mortality Worldwide for 36 Cancers in 185 Countries. CA Cancer J Clin. 2018;68(6):394–424. doi: 10.3322/caac.21492 30207593

[2] Wang Y-H, Song Z, Hu X-Y, Wang H-S. Circulating tumor DNA analysis for tumor diagnosis. Talanta. 2021;228:122220. doi: 10.1016/j.talanta.2021.122220 33773726

[3] Tian S. Identification of monotonically differentially expressed genes for non-small cell lung cancer. BMC Bioinformatics. 2019;20(1):177. doi: 10.1186/s12859-019-2775-8 30971213 PMC6458730

[4] Tian S, Wang C, Tang M, Li J, Liu W. Identification of Monotonically Differentially Expressed Genes across Pathologic Stages for Cancers. J Oncol. 2020;2020:8458190. doi: 10.1155/2020/8458190 33273919 PMC7676961

[5] Shi G, Wang Y, Zhang C, Zhao Z, Sun X, Zhang S, et al. Identification of genes involved in the four stages of colorectal cancer: Gene expression profiling. Mol Cell Probes. 2018;37:39–47. doi: 10.1016/j.mcp.2017.11.004 29179987

[6] Sarathi A, Palaniappan A. Novel significant stage-specific differentially expressed genes in hepatocellular carcinoma. BMC Cancer. 2019;19(1):663. doi: 10.1186/s12885-019-5838-3 31277598 PMC6612102

[7] Kim S, Nam H, Lee D. Exploring molecular links between lymph node invasion and cancer prognosis in human breast cancer. BMC Syst Biol. 2011;5:S4. doi: 10.1186/1752-0509-5-S2-S4 22784575 PMC3287484

[8] Peng W-X, Koirala P, Mo Y-Y. LncRNA-mediated regulation of cell signaling in cancer. Oncogene. 2017;36:5661–7. doi: 10.1038/onc.2017.184 28604750 PMC6450570

[9] Xing F, Liu Y, Wu S-Y, Wu K, Sharma S, Mo Y-Y, et al. Loss of XIST in Breast Cancer Activates MSN-c-Met and Reprograms Microglia via Exosomal miRNA to Promote Brain Metastasis. Cancer Res. 2018;78:4316–30. doi: 10.1158/0008-5472.CAN-18-1102 30026327 PMC6072593

[10] Soudyab M, Iranpour M, Ghafouri-Fard S. The Role of Long Non-Coding RNAs in Breast Cancer. Arch Iran Med. 2016;19:508–17. 27362246

[11] Safari MR, Mohammad Rezaei F, Dehghan A, Noroozi R, Taheri M, Ghafouri-Fard S. Genomic variants within the long non-coding RNA H19 confer risk of breast cancer in Iranian population. Gene. 2019;701:121–4. doi: 10.1016/j.gene.2019.03.036 30910558

[12] Cantile M, Di Bonito M, Cerrone M, Collina F, De Laurentiis M, Botti G. Long Non-Coding RNA HOTAIR in Breast Cancer Therapy. Cancers (Basel). 2020;12(5):1197. doi: 10.3390/cancers12051197 32397382 PMC7281113

[13] Pawłowska E, Szczepanska J, Blasiak J. The Long Noncoding RNA HOTAIR in Breast Cancer: Does Autophagy Play a Role? Int J Mol Sci. 2017;18 (11):2317. doi: 10.3390/ijms18112317 29469819 PMC5713286

[14] Muzio G O’Bray L, Borgwardt K. Biological network analysis with deep learning. Brief Bioinform. 2021;22:1515–30. 33169146 10.1093/bib/bbaa257 PMC7986589

[15] Berrar D, Dubitzky W. Deep learning in bioinformatics and biomedicine. Brief Bioinform. 2021;22:1513–4. doi: 10.1093/bib/bbab087 33693457 PMC8485073

[16] Ching T, Himmelstein DS, Beaulieu-Jones BK, Kalinin AA, Do BT, Way GP, et al. Opportunities and obstacles for deep learning in biology and medicine. 2018. doi: 10.1098/rsif.2017.0387 29618526 PMC5938574

[17] Xie R, Wen J, Quitadamo A, Cheng J, Shi X. A deep auto-encoder model for gene expression prediction. BMC Genomics. 2017;18 Suppl 9:845. doi: 10.1186/s12864-017-4226-0 29219072 PMC5773895

[18] Way GP, Greene CS. Extracting a biologically relevant latent space from cancer transcriptomes with variational autoencoders. Pac Symp Biocomput. 2018;23:80–91. 29218871 PMC5728678

[19] Li Y, Huang C, Ding L, Li Z, Pan Y, Gao X. Deep learning in bioinformatics: Introduction, application, and perspective in the big data era. Methods. 2019;166:4–21. doi: 10.1016/j.ymeth.2019.04.008 31022451

[20] Svensson V, Gayoso A, Yosef N, Pachter L. Interpretable factor models of single-cell RNA-seq via variational autoencoders. Bioinformatics. 2020;36:3418–21. doi: 10.1093/bioinformatics/btaa169 32176273 PMC7267837

[21] Lopez R, Regier J, Cole MB, Jordan MI, Yosef N. Deep generative modeling for single-cell transcriptomics. Nat Methods. 2018;15:1053–8. doi: 10.1038/s41592-018-0229-2 30504886 PMC6289068

[22] Li J, Han L, Roebuck P, Diao L, Liu L, Yuan Y, et al. TANRIC: An interactive open platform to explore the function of lncRNAs in cancer. Cancer Res. 2015;75:3728–37. doi: 10.1158/0008-5472.CAN-15-0273 26208906 PMC4573884

[23] Benjamini Y, Hochberg Y. Controlling the False Discovery Rate: A Practical and Powerful Approach to Multiple Testing. J R Stat Soc Ser B. 1995;57:289–300.

[24] Bao Z, Yang Z, Huang Z, Zhou Y, Cui Q, Dong D. LncRNADisease 2.0: an updated database of long non-coding RNA-associated diseases. Nucleic Acids Res. 2019;47:D1034–7. doi: 10.1093/nar/gky905 30285109 PMC6324086

[25] Yang F, Lyu S, Dong S, Liu Y, Zhang X, Wang O. Expression profile analysis of long noncoding RNA in HER-2-enriched subtype breast cancer by next-generation sequencing and bioinformatics. Onco Targets Ther. 2016;9:761–72. doi: 10.2147/OTT.S97664 26929647 PMC4758788

[26] Wang M, Liu H, Wu W, Zhao J, Song G, Chen X, et al. Identification of Differentially Expressed Plasma lncRNAs As Potential Biomarkers for Breast Cancer. Clin Breast Cancer. 2022;22(2):e135–e141. doi: 10.1016/j.clbc.2021.05.003 34119428

[27] Jonsson P, Coarfa C, Mesmar F, Raz T, Rajapakshe K, Thompson JF, et al. Single-Molecule Sequencing Reveals Estrogen-Regulated Clinically Relevant lncRNAs in Breast Cancer. Mol Endocrinol. 2015;29:1634–45. doi: 10.1210/me.2015-1153 26426411 PMC4627604

[28] Franceschini A, Szklarczyk D, Frankild S, Kuhn M, Simonovic M, Roth A, et al. STRING v9.1: protein-protein interaction networks, with increased coverage and integration. Nucleic Acids Res. 2013;41 Database issue:D808–15. doi: 10.1093/nar/gks1094 23203871 PMC3531103

[29] de Heer EC, Jalving M, Harris AL. HIFs, angiogenesis, and metabolism: Elusive enemies in breast cancer. J Clin Invest. 2020;130:5074–87. doi: 10.1172/JCI137552 32870818 PMC7524491

[30] Gong L, Tang H, Luo Z, Sun X, Tan X, Xie L, et al. Tamoxifen induces fatty liver disease in breast cancer through the MAPK8/FoxO pathway. Clin Transl Med. 2020;10:137–50. doi: 10.1002/ctm2.5 32508033 PMC7240857

[31] Xu X, Zhang M, Xu F, Jiang S. Wnt signaling in breast cancer: biological mechanisms, challenges and opportunities. Mol Cancer. 2020;19(1):165. doi: 10.1186/s12943-020-01276-5 33234169 PMC7686704

[32] Koni M, Pinnarò V, Brizzi MF. The Wnt signalling pathway: A tailored target in cancer. Int J Mol Sci. 2020;21(20):7697. doi: 10.3390/ijms21207697 33080952 PMC7589708

[33] Yin P, Wang W, Zhang Z, Bai Y, Gao J, Zhao C. Wnt signaling in human and mouse breast cancer: Focusing on Wnt ligands, receptors and antagonists. Cancer Sci. 2018;109:3368–75. doi: 10.1111/cas.13771 30137666 PMC6215866

[34] Wang T, Chang P, Wang L, Yao Q, Guo W, Chen J, et al. The role of human papillomavirus infection in breast cancer. Med Oncol. 2012;29:48–55. doi: 10.1007/s12032-010-9812-9 21318737

[35] Khodabandehlou N, Mostafaei S, Etemadi A, Ghasemi A, Payandeh M, Hadifar S, et al. Human papilloma virus and breast cancer: The role of inflammation and viral expressed proteins. BMC Cancer. 2019;19(1):61. doi: 10.1186/s12885-019-5286-0 30642295 PMC6332859

[36] Zhang K, Wang Y-W, Wang Y-Y, Song Y, Zhu J, Si P-C, et al. Identification of microRNA biomarkers in the blood of breast cancer patients based on microRNA profiling. Gene. 2017;619:10–20. doi: 10.1016/j.gene.2017.03.038 28359916

[37] Lee S-C, Xu X, Lim Y-W, Iau P, Sukri N, Lim S-E, et al. Chemotherapy-induced tumor gene expression changes in human breast cancers. Pharmacogenet Genomics. 2009;19:181–92. doi: 10.1097/FPC.0b013e32831ebb5d 19352302

